# Learning Set Formation and Reversal Learning in Mice During High-Throughput Home-Cage-Based Olfactory Discrimination

**DOI:** 10.3389/fnbeh.2021.684936

**Published:** 2021-06-09

**Authors:** Alican Caglayan, Katharina Stumpenhorst, York Winter

**Affiliations:** ^1^Institute for Biology, Humboldt Universität, Berlin, Germany; ^2^Neurocure, Charité – Universitätsmedizin Berlin, Berlin, Germany

**Keywords:** cognition, automated behavioral analysis, sorting system, learning set, reversal learning

## Abstract

Rodent behavioral tasks are crucial to understanding the nature and underlying biology of cognition and cognitive deficits observed in psychiatric and neurological pathologies. Olfaction, as the primary sensory modality in rodents, is widely used to investigate cognition in rodents. In recent years, automation of olfactory tasks has made it possible to conduct olfactory experiments in a time- and labor-efficient manner while also minimizing experimenter-induced variability. In this study, we bring automation to the next level in two ways: First, by incorporating a radio frequency identification-based sorter that automatically isolates individuals for the experimental session. Thus, we can not only test animals during defined experimental sessions throughout the day but also prevent cagemate interference during task performance. Second, by implementing software that advances individuals to the next test stage as soon as performance criteria are reached. Thus, we can prevent overtraining, a known confounder especially in cognitive flexibility tasks. With this system in hand, we trained mice on a series of four odor pair discrimination tasks as well as their respective reversals. Due to performance-based advancement, mice normally advanced to the next stage in less than a day. Over the series of subsequent odor pair discriminations, the number of errors to criterion decreased significantly, thus indicating the formation of a learning set. As expected, errors to criterion were higher during reversals. Our results confirm that the system allows investigating higher-order cognitive functions such as learning set formation (which is understudied in mice) and reversal learning (which is a measure of cognitive flexibility and impaired in many clinical populations). Therefore, our system will facilitate investigations into the nature of cognition and cognitive deficits in pathological conditions by providing a high-throughput and labor-efficient experimental approach without the risks of overtraining or cagemate interference.

## Introduction

Olfaction is a primary sensory modality for rodents. They learn olfactory stimulus–reward associations more readily than associations involving visual or auditory stimuli (Nigrosh et al., 1975). As a result, olfactory stimuli are highly suitable to investigate cognitive functions such as reversal learning (Eichenbaum et al., 1986; Schoenbaum et al., 2002), working memory (Winters et al., 2000; Kesner et al., 2011) and attentional set shifting (Birrell and Brown, 2000; Scheggia et al., 2014; reviewed in Slotnick, 2001). Of special interest in the context of cognitive functions is the phenomenon of learning set formation, which was first described in primates and children as the progressive improvement in performance after successive training on similar problems (Harlow, 1949). Underlying such improvement may be a transfer across problems resulting in the acquisition of a response rule such as “win–stay, lose–shift,” which makes learning set formation a higher-order cognitive function (Levine, 1959). Still, rodents’ ability to acquire a learning set through rule acquisition akin to primates has also been questioned in favor of more parsimonious explanations for performance improvements, such as the abandonment of inefficient response tendencies with extensive training (Reid and Morris, 1992, 1993). However, after multiple discrimination problems, rats display strikingly higher performance than after similar training on just one discrimination problem (Slotnick et al., 2000). This finding supports the presence of learning set formation and the acquisition of higher-order response rules even in rats. Further evidence of learning set formation in rodents comes from the identification of dissociable neural structures that may be involved (Whishaw, 1987; Lu and Slotnick, 1990; Bailey et al., 2003; Compton, 2004).

Another cognitive function of interest here is cognitive flexibility, which underlies performance in reversal learning. Reversal learning is a special case of discrimination learning in which reward contingencies between a previously rewarded stimulus and an unrewarded stimulus are switched. As reversal learning requires adapting to new reward contingencies and inhibiting pre-potent responses to previously rewarded stimuli, it is considered to be a measure of cognitive flexibility. Deficits in reversal learning are observed in many neurological and psychiatric conditions, such as schizophrenia (Waltz and Gold, 2007; Schlagenhauf et al., 2014), dementia (Freedman and Oscar-Berman, 1989; Rahman et al., 1999), depression (Robinson et al., 2012) and addiction (Ersche et al., 2011). This association with neurological and psychiatric conditions makes reversal learning in rodents a translationally relevant task.

The goal of the present study is to improve the methodology available to experimentally investigate cognition and cognitive dysfunction in mouse models. Despite a growing understanding of the mechanisms underlying cognitive functions, the current classification of psychiatric diseases is still mostly symptom based. Replacing this classification system with one based on pathophysiology will require many more animal model studies. When higher cognitive functions are targeted and a potentially complex and laborious training of the mice is involved, progress will depend on the availability of highly efficient methods for mouse behavioral training and testing. Such methods require fully computer-automated procedures, which are the topic of this study.

Olfaction-based behavioral experiments have often been carried out manually, which requires experimenter involvement at every step (Berger-Sweeney et al., 1998; Mihalick et al., 2000; Rushforth et al., 2010). Nowadays, computers control odor stimulus presentation, reward delivery and data acquisition (Slotnick and Risser, 1990; Bodyak and Slotnick, 1999; Larson and Sieprawska, 2002; Abraham et al., 2004; Qiu et al., 2014). A significant recent advance are experiments taking place in the home cage (Erskine et al., 2019; Reinert et al., 2019). Home cage-based studies decrease not only workload but also experimenter-induced data variability by eliminating the need to move animals to the operant chamber for each session (Galsworthy et al., 2005; de Visser et al., 2006; Maroteaux et al., 2012; Balci et al., 2013). Such variability is considered a contributing factor to observed inter-laboratory variance in rodent behavioral experiments (Chesler et al., 2002; Sorge et al., 2014), and thus data obtained via home-cage-based experimentation systems have low inter-laboratory variance (Lipp et al., 2005; Krackow et al., 2010; Endo et al., 2011).

One important distinction is whether such a system allows the researcher to group-house animals (Galsworthy et al., 2005; Knapska et al., 2006; Endo et al., 2011; Dere et al., 2018; de Chaumont et al., 2019) or if it requires the isolation of animals (Poddar et al., 2013; van Dam et al., 2013; Remmelink et al., 2016, 2017). Careful consideration must always precede the experimental design as group housing can lead to the formation of dominance relationships and aggression and may introduce asymmetric variation if different treatment groups are housed together (Blanchard et al., 1988; Kappel et al., 2017). Long-term social isolation on the other hand induces negative behavioral changes in rodents (van Loo et al., 2003; Arndt et al., 2009; Martin and Brown, 2010). Without contraindication group housing is therefore generally viewed as preferable, as it also allows for multiple animals to be tested in one system. Group-housed animals are commonly marked with subcutaneous ID chips (radio frequency identification [RFID] transponders) to allow individual experimentation.

In this study, we used an automated olfactory task and a home cage with a group of ID-chipped mice. We then connected the two compartments by an RFID-based animal sorter. This sorter allowed continuous testing throughout the 24-h period by giving mice individual access to the test compartment automatically. Experimental sessions were thus self-initiated and voluntary as well as free from the interference of cagemates. As shown previously, rodents readily adapt to the animal sorting process with minimal sorter training (Winter and Schaefers, 2011; Rivalan et al., 2017).

The experimental efficiency of our system further benefited from our implementation of immediate performance-based advancements in the behavioral schedule. Once a performance level was reached, an animal advanced to the next experimental stage on the same day, or even within the same experimental session, and without experimenter involvement. Such a feature has not been implemented in other home-cage-based olfactory testing systems. It saves experimental time by advancing the animal more quickly through the stages of a behavioral schedule. It also prevents overtraining, which might affect reversal learning (Machkintosh, 1962; Dhawan et al., 2019).

In this study we evaluated the potential of our system for research on cognitive functions by training mice on a series of two-odor discriminations with four odor pairs. This included initial acquisition of an odor pair and its subsequent reversal and allowed us to evaluate (i) learning set formation (i.e., the progressive improvement in task performance with each subsequent discrimination) and (ii) reversal learning (i.e., the expected decrease in performance during the reversal learning stage). Both are measures of higher cognitive functions.

We observed learning set formation and the expected performance decrease during reversal learning, which demonstrates the suitability of our approach for studying higher-order cognitive functions. Furthermore, the high degree of automation allowed fast, high-throughput and labor-efficient experimentation with minimal experimenter involvement.

## Methods

### Animals

Twelve C57BL/6JRj male mice (Charles River, Germany) aged 8 weeks were housed in groups of six in standard EU type III cages (43 × 27 × 18 cm). Prior to study onset, they have received biocompatible RFID transponders (12.1 mm × 2.1 mm, Sokymat, Switzerland). Animals were kept on a 12 h light/12 h dark cycle at 23 ± 2°C and 45–55% rel. humidity in the experimental chamber, to which they were transferred 6 days prior to the start of the experiment for chamber habituation. Experiments were carried out with two groups of six animals in succession. Maintenance chow (V1535, Ssniff, Germany) was provided *ad libitum* throughout the experiment. During the chamber habituation period, water was provided from a bottle in the home cage. During the experimental phase, water was provided from the liquid feeder in the operant chamber. Water consumption was monitored daily, and mice that had drunk less than 1 ml received 30 min of access to a water bottle in a separate home cage. Furthermore, a daily visual inspection was performed on all mice.

### Ethics

All procedures were conducted in compliance with the European Communities Council Directive 2010/63/EU and under the supervision and with the approval of the animal welfare officer at Humboldt University. Generally, our approach aims to maximize welfare by using undisturbed home-cage-based experimentation. Due to the study’s observational nature, the animals did not experience damage, pain or suffering.

### Apparatus

**FIGURE 1:**
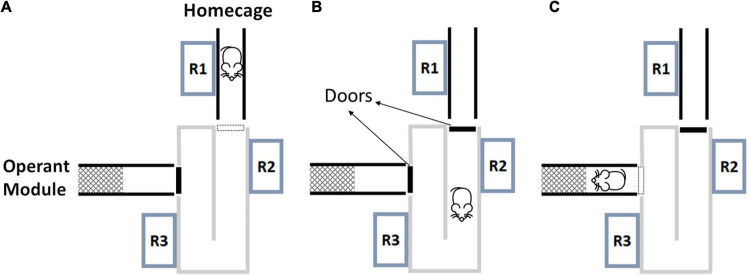
The animal sorter and the sorting process. The sorter connects the home cage with the operant module. **(A)** A mouse detected at R1 triggers the opening of door 1. **(B)** Door 1 closes again once the mouse is detected at R3. The mouse remains between closed doors for 30 s. R2 and R3 are used to verify that only a single mouse is inside the sorter (verification interval). **(C)** After single entry is verified, door 2 opens and releases the mouse to the operant module. R, radio frequency identification reader (mouse icon from Selman Design, CC BY).

The experimental system consisted of a home cage, an animal sorter (ID Sorter, PhenoSys, Germany) and the operant module with odor stimulation (Knosys olfactometers, United States). The software PhenoSoft Control (PhenoSys, Germany) controlled the system from a PC. Animals could enter the RFID-based sorter through a Plexiglas tube from their home cage. The basic principle of the animal sorter system has been described elsewhere (Winter and Schaefers, 2011). Briefly, it consisted of a U-shaped tunnel with a motor-controlled guillotine door at each end and three RFID readers to detect and identify a mouse (Figure 1). If a mouse was identified at RFID reader 1, then door 1 opened and the animal entered the sorter. Once the animal was registered at reader 3, door 1 closed. Thereafter, the animal remained within the sorter compartment for 30 s. During this interval, data from readers 2 and 3 were used to verify that only a single animal was within the sorter compartment, and if so door 2 opened and released the animal to the operant module. If a second mouse was detected, door 1 reopened and the sorting process was reinitialized. This sorting procedure worked reliably in the present study and animals were never observed to get stuck in the process. In a recent version of the ID sorter, not yet used in the current work, the sorting chamber always rests on a laboratory balance. As the weight immediately indicates the presence of multiple mice, this considerably speeds up the sorting process.

**FIGURE 2:**
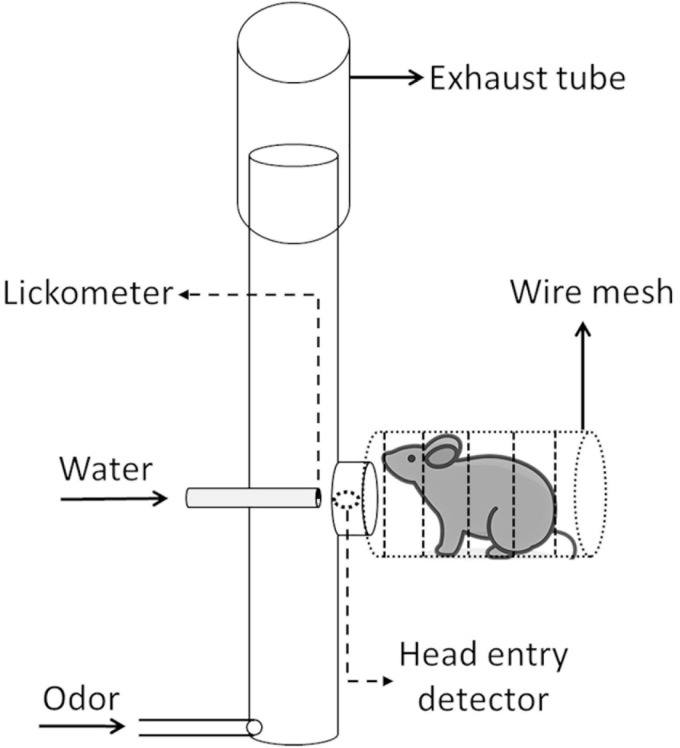
Setup and function of the operant module. A mouse positions itself in a wire mesh tube in front of the operant module head entry port. A photoelectric head entry detector detects any head insertion. A head insertion can trigger an odor stimulus release (odor generator not shown). Upon odor sampling, the mouse can then lick at the 13-gauge stainless-steel waterspout, thus signaling a “go” response. Licks are detected by a sensor jointly connected to the wire mesh tube and lick spout. Upon licks, a water reward may be released from the spout. A constant carrier airflow from bottom to top carries any injected odor stimulus through the glass tube to the nose of the mouse and then removes it through the exhaust tube (mouse icon by Vincent Le Moign, CC BY).

The operant module functioned as an olfactory stimulus delivery port, a response sensor and a reward delivery port. From the mouse’s point of view, this was a small tubular compartment consisting of a wire mesh tube (3 cm diameter, 6.5 cm long) with a head entry opening at the far end (Figure 2). Through this opening the mouse had access to the water delivery port and could also sample the odor in the airstream passing the tube.

**FIGURE 3:**
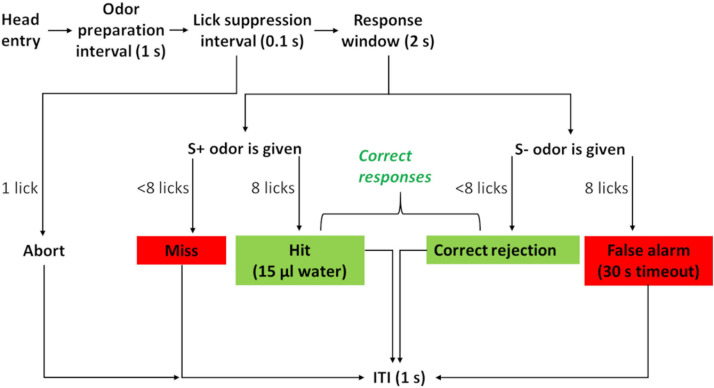
Flowchart of a trial during the two-odor discrimination task. Head entry triggers internal olfactometer odor release. During odor preparation, the odor gas fills the tubing up to the operant module. Odor is released to the operant module from the beginning of the lick suppression interval. Any lick starting during this interval leads to abortion of the trial. This ensures that a mouse actually perceives the odor before responding. Licks during the response window are counted, and eight licks to the S+ odor are rewarded (hit). A positive response to the S− odor (false alarm) is negatively reinforced by timeout. Not responding to S+ odor (miss) or S− odor (correct rejection) leads to an ITI (inter-trial-interval). Correct responses are green; incorrect responses are red.

Odor stimuli were generated and delivered through a Knosys olfactometer system with eight odor channels (Bodyak and Slotnick, 1999). Briefly, PET bottles (polyethylene terephthalate, 240 ml, Container and Packaging Supply, United States) served as odor saturator bottles, which were connected through C-flex tubing (6 mm OD, Cole Parmer, United States) to the operant module. Airflow was controlled by pinch valves. During the 1-s odor preparation interval (Figure 3), an odor was flushed to the final delivery valve, where it was initially diverted to exhaust. Only upon stimulus onset did the final valve switch the odor to flow to the odor stimulus port. This presence of the final valve ensured that the timing of odor presentation was the same for all odors since the lengths of connecting tubes differed between bottles. The initial 100 ms after the onset of odor presentation was the lick suppression interval. Licks starting during the lick suppression interval aborted the trial. This approach ensured that mice perceived the odor stimulus before responding by licking.

### Behavioral Procedure

#### Task Training

**FIGURE 4:**
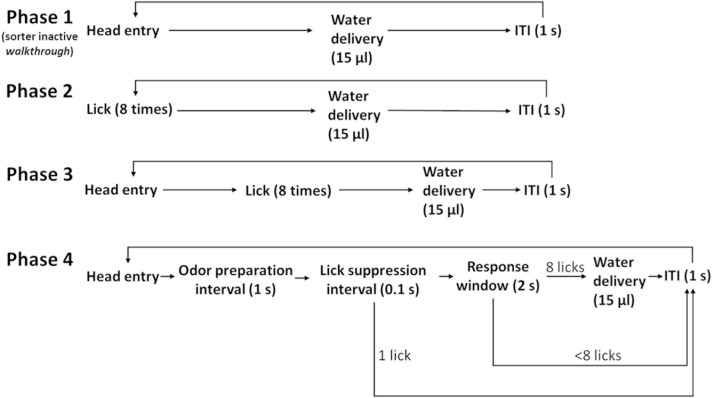
Flow chart of the training procedure. Mice learn, in phase one, to obtain water from the operant module at the end of the sorter; in phase two, to lick as a response to trigger water delivery; in phase three, to re-enter their head to keep initiating trials; and in phase four, to accustom themselves to odor valve clicking and suppress early licking. Mice trained in 3 days (except for one). The ITI was set to the minimal value of 1 s.

Mice went through four phases of training (Figure 4), which took a total of 3 days (except one mouse which took 4 days). In phase one, the sorter was open and served just as a walkthrough tunnel to the operant module. Mice learnt to obtain water from the waterspout. Each separate head entry was rewarded with 15 μl of water. Phase one lasted 1 day. From phase two, the sorting procedure was activated. After operant module entry, a session lasted 30 min. After exit, an individual was not allowed to re-enter for at least 1 h (minimal inter-session interval). In phase two, mice learnt to lick repetitively. A water reward was given after each set of eight licks. Phase two lasted 1 day. In phase three, mice also received a water reward for every eight licks. However, head retraction and head re-entry were required between rewards. Phase three lasted until a mouse had obtained 50 rewards.

During phase four, mice became accustomed to the clicking of the odor valves and learnt to suppress licking during the initial 1.1 s when an odor stimulus would not yet be present. After head entry, odor valves clicked immediately, and they did so again after 1.0 s with audible clicks. Critical was the “lick suppression interval,” which was the 100-ms interval from 1,000 to 1,100 ms after head entry. Licks starting during this interval led to an aborted trial. This process taught mice to refrain from licking early. Instead, they learnt to lick only when a putative odor stimulus would also be perceivable. Despite odor valve operation, no odors were delivered during phase four. This phase was completed after 50 successful trials. The two-odor discrimination experiment then started on the next day.

#### Discrimination Task

During our experiment, mice went through a series of four two-odor pair discriminations. These were presented as an initial acquisition that was directly followed by a stimulus reversal, where the S+ odor became the S- odor, and vice versa (Table 1). Odor discrimination trials proceeded as the previous training phase four, now including odor stimulus delivery. Individual sessions lasted 30 min, re-entry after a session was blocked for 1 h, and the ITI (inter-trial-interval) remained at the minimum of 1 s. Mice had to respond to the positive stimulus with a “go” response (eight licks within 2 s), a “hit.” Accordingly, they had to suppress their response to the negative odor stimulus with a no-go response (fewer than eight licks within 2 s), considered a “correct rejection.” This no-go response toward the S- odor stimulus was a novel behavioral requirement of the discrimination task that had not been taught previously. A go response to an S- odor (“false alarm”) was negatively reinforced by a 30-s timeout. Not responding led to the ITI, both for correctly rejecting an S- odor and for missing an S+ odor. All hits and correct rejections were counted as correct responses (Figure 3).

**TABLE 1:** Experimental sequence in eight stages with odor pairs in an exemplary order.

**Stage**	**Discrimination**
First initial acquisition	**Eucalyptol/**dihydrojasmone
First reversal	Eucalyptol/**dihydrojasmone**
Second initial acquisition	**Methyl salicylate**/α-ionone
Second reversal	Methyl salicylate/**α -ionone**
Third initial acquisition	**Limonene**/ethyl lactate
Third reversal	Limonene**/ethyl lactate**
Fourth initial acquisition	**Anisole**/eugenol
Fourth reversal	Anisole/**eugenol**

*The rewarded S+ odor is shown in bold.*

The sequence of odor pairs and the initial S+ odor werepseudo-randomly assigned to the mice for counterbalancing. We firstcreated a 4 × 4 Latin square for all odor pairs across thenumber of discriminations and then replicated this Latin square withcontingencies reversed between S+ and S-. Therefore, if an animal had S+ anisole during the third initial acquisition, another animal had S+ eugenol during the third initial acquisition (from the anisole/eugenol pair). As we had 12 subjects, we needed two additional random sequences and their counter-balanced sequences. As one mouse that did not learn was excluded from the analysis, the data shown are for 11 mice. Both the initial acquisition and reversal stages ended when performance reached the criterion of 85% correct responses in 20 consecutive trials. The experimental switch to the next stage (reversal or next odor pair) occurred within ongoing sessions. We implemented this performance-based stage switching in the experimental control software so that it occurred automatically. Otherwise, as commonly done, a mouse could have advanced to the next experimental stage only on the next experimental day. This would have significantly extended the duration of the whole experiment. Also, maintaining training after the criterion is reached could lead to overtraining which may impact later training stages. The experiment ended for a mouse when it had completed all eight stages of odor discrimination learning and reversal. After finishing the experiment, the mouse stayed in the system and was re-started on its discrimination series until all the other mice had completed the experiment.

### Odors

The eight odors were presented in four fixed pairs: (1) anisole–eugenol, (2) α- ionine–methyl salitate, (3) ethyl lactate–lemonine, and (4) dihydrojasmone–eucalyptol. Odors were obtained from Sigma Aldrich (Munich, Germany). All odorant liquids were used as undiluted pure substances. We did not dilute odor substances to equilibrate for equal vapor partial pressure or salience between odors. However, as we randomized and balanced our treatments across all odor this should not affect our presented results. Odor bottles were filled with 20–50 ml liquid. Two silicon tubes were inserted into each bottle. The air pump pressed air through the inlet tube into the odorant liquid to ensure odor saturation (Slotnick and Restrepo, 2005). The odorized air from the head space then left through the outlet to the operant module. During odor presentation, airflow through the respective odorant bottle was 0.05 l/min. Odorized air was then diluted with 1.95 l/min of clean air (Bodyak and Slotnick, 1999). As the odor liquids were undiluted in the bottles, the final stimulus was 2.5% saturated odorant vapor. Valves in this setup make a click noise as they activate specific individual odors. Mice should only respond to the odors and not recognize and discriminate between valve clicks. The necessary control experiment to show that in the presence of clicks but absence of odors mice remain at chance level performance has been demonstrated previously using the same model olfactometer (Bodyak and Slotnick, 1999).

### Statistical Analysis

Number of errors to criterion (85% correct responses in 20 consecutive trials) was used as the performance measure. To normalize the distribution, number of errors was log transformed. A random intercept linear mixed effect model was fitted to the log-transformed data using *number of discriminations* and *contingency* (initial acquisition vs. reversal) as fixed factors and *subject* as a random effect. Since the interaction term number of discriminations and contingency did not reach statistical significance, it was not included. Furthermore, the model without the interaction term was a better-fit according to AIC (the Akaike information criterion [AIC] was higher than in the model without interaction, AIC_Δ_ = 4.99). Data analysis and visualization were performed using R 4.0.2 (R Core Team, 2020). Model fitting used the lme4 package (Bates et al., 2015), and degrees of freedom and *p*-values were calculated with the lmerTest package (Kuznetsova et al., 2017). Box plots show medians, 1st and 3rd quartiles, whiskers represent the 1.5 interquartile range.

## Results

### Sorter and Task Training

With the beginning of the first day of training mice entered the sorter within 5 min after all doors had opened (median = 4.6 min, max. 12 min). For the rest of the first day, still without sorter functionality but with doors always open, mice entered the operant compartment and made an average of 180 individual head entries, collecting 2.7 ml (mean) of water.

The sorter was activated from phase two onwards. During training phases, mice were sorted into the operant compartment for between 2 and 9 sessions per day (median 5 sessions) and the operant module was occupied for nearly 14 h per day with mice performing sessions. Mice, especially at the beginning, tended to crowd together in the sorter, which led to the sorting procedure being aborted. Thus, for each successful visit to the operant compartment, a mouse needed an average of 5 entries to the sorter. A mouse completed 122 trials on average per day (98–167 trials). The minimum of 50 trials that were required for each of the training phases three and four were completed within a single day by all mice except one.

### Odor Pair Discrimination Acquisition and Reversal

**FIGURE 5:**
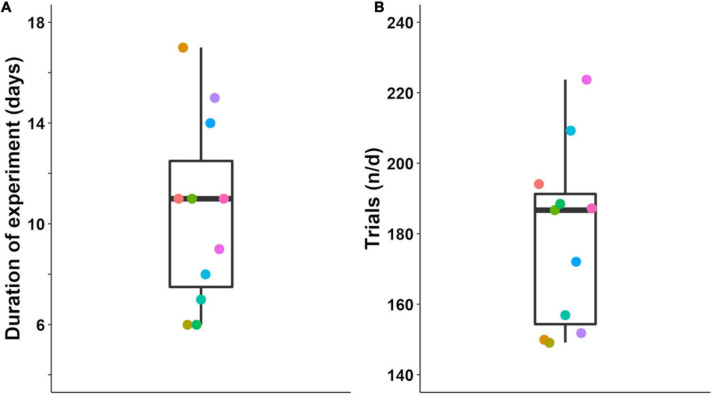
Time required for the acquisition of four successive odor pair discriminations and their reversals. **(A)** Days to complete all four acquisitions and reversals. Stage-criterion was 85% correct in 20 successive trials. **(B)** Mean number of trials per day, n, total number of trials; d, number of days. Colored dots represent data from the same individuals in **(A,B)**; box plots show median, first and third quartiles, and whiskers indicate the 1.5 interquartile range. Data from *n* = 11 mice.

Mice completed the four initial odor pair discrimination acquisitions and their respective reversals in 6–17 days (median 11 days, Figure 5A). On average, they completed 149–224 individual trials per day (median 187, Figure 5B). When a mouse reached the criterion for a stage, it was advanced to the next stage (reversal or next odor pair) immediately—that is, within the same session. This performance-based advancement in the experimental schedule was conducted automatically by the software. If mice had to wait until the next day to advance in their task schedule, the four acquisition and reversal stages would have lasted 9–21 days (median 14 days)—in other words, 3–4 days longer.

**FIGURE 6:**
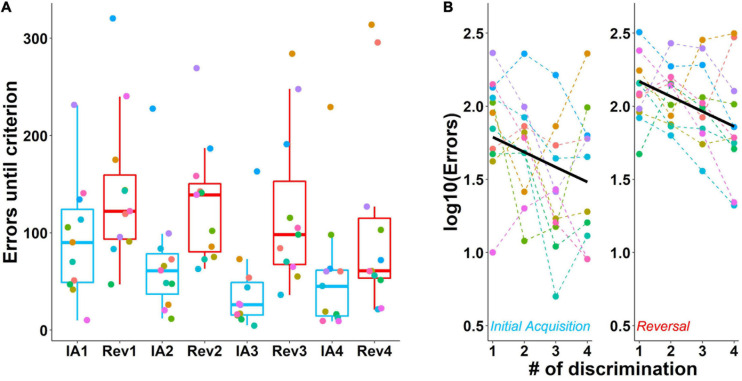
Performance during the acquisition of four successive odor pair discriminations and their reversals. **(A)** Number of errors until achievement of the criterion during the initial acquisition of an odor pair (blue) and its reversal (red). Mice made more errors during reversals. **(B)** Rearranged data from A with thick lines showing the model predictions. The number of errors decreased with each subsequent discrimination. Data are log-transformed to show model predictions as a straight line. Colored dots connected by dashed lines show individual mice. Box plots show median, first and third quartiles, and whiskers the 1.5 interquartile range. IA, initial acquisition, Rev, reversal. Criterion was 85% correct in 20 trials. Data from *n* = 11 mice.

More errors were made during reversals compared to the initial acquisition stages (Figure 6A). Furthermore, errors decreased across discrimination stages for both initial acquisition and subsequent reversals. Statistical analysis of the log-transformed data confirmed this effect of contingency on performance from initial acquisition to the reversal stage [*F*_(__1_, _75__)_ = 37.29, *p* < 0.001] and also the effect of number of discriminations on performance [*F*_(__1_, _75__)_ = 13.46, *p* < 0.001, Figure 6B]. In Figure 6 the slope of a line shows the influence of the number of discriminations that a mouse has experienced. The difference in the intercepts of the lines shows the effects of the two experimental contingencies, here, initial acquisition and reversal stage. Along with *p*-values, we also report confidence intervals (Supplementary Figure 1).

To provide an overview of session distributions and stage progressions for each individual animal in the automated system, we plotted the duration of stages and start times of each session against the timeline of the experiment (Supplementary Figure 2). Furthermore, we visually investigated how the time of day was correlated with the number of trials performed within the session and with performance during the sessions (Supplementary Figure 3). Although performance during the sessions did not correlate with the time of day, it appears that during certain time bins within the dark cycle, mice performed more trials within sessions. However, we found no visible correlation between number of trials performed in a session and performance during the session (Supplementary Figure 4).

## Discussion

In this study we developed and validated an automated home-cage-based system to investigate cognitive function in mice using odor stimuli. Single individuals from group-housed mice voluntarily entered the operant module through an RFID-based sorter system to perform an odor discrimination task without cagemate interference. Once animals reached the criterion in one stage, they automatically proceeded to the next test stage within the same session, thus avoiding overtraining. Mice successfully completed four successive odor pair discriminations as well as their reversals within 6–17 days and showed hallmarks of learning set formation. Compared to conventional systems, which commonly require animal handling and water restriction before each session (Bodyak and Slotnick, 1999), our system minimizes experimenter-induced variability while increasing labor efficiency and animal welfare.

Analysis of mouse performance during subsequent odor discriminations indicated that animals not only successfully learnt to discriminate each odor pair but also formed a learning set, as the median number of errors between the first and forth odor pair discrimination acquisition halved. Very few studies have investigated olfactory learning set formation in mice to date; while one study reported a 70% decrease in errors between the first and second discrimination (Larson et al., 2003), two other studies observed effect sizes similar to those reported here (Larson and Sieprawska, 2002; Patel and Larson, 2009).

Compared to the initial acquisition stages, a hallmark of reversal learning is that animals make more errors, an effect that is caused by the additional difficulty of inhibiting the previously correct response (Dias et al., 1996; Clark et al., 2004). In the present study, the median number of errors increased by 36–277% depending on the reversal stage. The magnitude of this effect was similar to previous olfactory reversal learning studies in mice (Mihalick et al., 2000; Kruzich and Grandy, 2004) and also comparable to studies in which compound stimuli included odor as a dimension (Colacicco et al., 2002; Garner et al., 2006).

The RFID-based sorter is an integral part of the system as it enables access of a single identified mouse to the operant module. All mice readily explored the novel sorter environment within a couple of minutes and quickly learnt to use the sorter to reach the operant module. Furthermore, by voluntarily initiating approximately five sessions per day, all except one mouse were able to complete the four training phases within 3 days. Performance during the olfactory discrimination and reversal stages was quite variable between mice. While some animals completed all eight stages within 6 days, one mouse required 17 days. The learning rates that we observed are in line with previous findings (Mihalick et al., 2000; Reinert et al., 2019) in both automated and conventional experimental setups. Therefore, these individual differences in learning rates might well reflect typical variance in individual behavior, as we did not observe a correlation between the days to criterion and the mean trial number per day.

One of the new features that allowed mice in our setup to complete several training phases or test stages within a single day was the introduction of performance-based advancement in the experimental schedule not only within a day but also within a session. This automated progression through the stages reduced the median completion time by 3 days across the 20-day study, an advantage that is only expected to become more pronounced with longer discrimination series. In addition, immediate performance-based advancements prevent overtraining at any given stage. This is relevant since overtraining affects measures of cognitive flexibility, such as reversal learning and attentional set shifting (Capaldi and Stevenson, 1957; Brookshire et al., 1961; Garner et al., 2006; Dhawan et al., 2019), and differences due to varying overtraining can be misinterpreted as differences in cognitive flexibility across the individuals. Especially in a home-cage-based setting in which the degree of overtraining could vary massively between animals that reach criterion during the first or last session of a day, we believe that automated performance-based advancement is essential.

Taken together, the results for our home-cage-based system are comparable to those observed in previous conventional experiments. Our system is therefore well suited to study cognitive functions such as learning set formation and cognitive flexibility during reversal learning with the added advantage of a high-throughput automated home-cage-based approach.

To our knowledge, there is only one other home-cage-based olfactory discrimination system to date in which socially housed mice can be tested individually. In the AutonoMouse setup (Erskine et al., 2019), individual mice enter the behavioral area through a door. IR (infrared) detectors within the behavioral area signal occupation and prevent further animals from accessing the door while also triggering RFID tag detection in the behavioral staging area (Erskine et al., 2019). As we used a more stringent learning criterion (85% instead of 80% correct responses), results cannot be compared directly. However, re-analysis of our data using the 80% correct criterion revealed that compared to Erskine et al. (2019), in which only two discriminations were tested, in our system the median number of errors was one-third higher during the first discrimination stage and more than twice as high during the second discrimination stage. Several differences between the two studies may have contributed to this finding. First and foremost, different odor pairs were used. This is relevant because odor salience has repeatedly been shown to affect odor discrimination performance (Slotnick and Katz, 1974; Slotnick et al., 2000; Chu et al., 2016). Furthermore, mice in the cited study (Erskine et al., 2019) did not automatically proceed to the next stage once performance criteria were reached. This continuation of training past the performance criterion may solidify the learning set and decrease the number of errors in subsequent discriminations. Although it could be argued that mice in the present study were trained to a higher performance criterion and thus also past the 80% criterion, mice generally needed less than 10 additional trials to reach this higher performance criterion, while in the cited study (Erskine et al., 2019) mice were trained on a few hundred more trials to reach asymptotic performance. Training past the 80% criterion was thus much more pronounced in Erskine et al. (2019) compared to our study. In addition, in the present study a reversal stage was included after each initial acquisition stage. However, reversals (especially serial reversals) have been shown to facilitate learning set formation (Schusterman, 1964; Warren, 1966) and may thus not contribute to the observed differences. Nonetheless, given the differences between the two studies, the relevant experimental factors contributing to the differences in results require further investigation.

One advantage both systems offer is the prevention of cagemate interference during task performance. While in the AutonoMouse setup a rush of several mice resulted on rare occasions in the entrance of more than one mouse before the door closed, this was never observed in our system due to the verification period during the sorting process. The elimination of cagemate interference is important since it can affect the behavior of a mouse during task performance. Apart from disturbing an animal and drawing attention away from the task, social interference can modulate learning and memory (Knapska et al., 2010; Nowak et al., 2013), though this effect has mainly been studied in fear-conditioning paradigms. Furthermore, social interference can influence access to the operant module, especially if there are large differences in dominance, if there are many animals per operant module or if there is increased aggression between cagemates (e.g., due to genotype) (Nelson and Chiavegatto, 2000; Endo et al., 2012). Where hierarchies and competition might affect or bias results, individuals or treatment groups could still be kept separate in multiple independent home cages with multiple sorters connecting these to one jointly used operant system.

An additional advantage of our sorter system is that it provides temporal control of the session duration as well as the inter-session interval. Because mice are not allowed to immediately re-enter the operant module after a session, other cagemates have an opportunity to gain access to the operant module. Inter-session intervals are furthermore able to reduce “pseudo-sessions” in which animals initiate a session without engaging in the task (Rivalan et al., 2017).

Our results show that our home-cage-based system is highly suitableto efficiently study learning set formation and behavioralflexibility. In the future, a similar setup may be used to studyattentional set shifting. In this task, animals learn to shift theirattention from one dimension (e.g., odor) of paired two-dimensionalstimuli to another (e.g., texture) (Birrell and Brown, 2000; Colacicco et al., 2002; McAlonan and Brown, 2003; Tanaka et al., 2011; Marquardt et al., 2014). Papaleo’sgroup developed a two chamber, computerized version of this task inwhich mice learnt the first initial discrimination within 30 min forolfactory, visual, and tactile discriminations alike (Scheggia et al., 2014). Such acquisition speed with mice has, to our knowledge, not been reached by any other setup. Deficits in attentional set shifting have been reported in several disorders, such as schizophrenia, attention deficit hyperactivity and obsessive-compulsive disorder (Elliott et al., 1995; Watkins et al., 2005; Rohlf et al., 2012). We believe that further automation of the attentional set-shifting task by incorporating it into our home-cage-based test system will be beneficial to further research in this area and may facilitate pre-clinical high-throughput drug discovery studies.

## Conclusion

In the present study, we demonstrated the potential of a home-cage-based olfactory discrimination system for studying cognitive functions by training mice on the initial discrimination and reversal of four odor pairs. We observed learning set formation as well as the expected increase in errors during reversals, thus proving that this system is well suited to study higher-order cognitive functions. Compared with other home-cage-based systems, our system especially benefits from temporally and spatially separated test sessions without cagemate interference, as well as the prevention of overtraining by automated and immediate performance-based advancement of individual mice through test stages. This system thus facilitates high-throughput, labor-efficient olfaction-based cognitive experiments with minimal experimenter involvement.

## Data Availability Statement

The raw data supporting the conclusions of this article will be made available by the authors, without undue reservation.

## Ethics Statement

The animal study was reviewed and approved by the Animal Welfare Officer of Humboldt University.

## Author Contributions

AC and YW conceived the project. AC conducted the experiment under the supervision of YW and KS. AC performed data analysis and prepared the figures. All authors contributed to writing of the manuscript.

## Conflict of Interest

YW owns PhenoSys equity. The remaining authors declare that the research was conducted in the absence of any commercial or financial relationships that could be construed as a potential conflict of interest.
