# Supplementary material for: Learning Set Formation and Reversal Learning in Mice During High-Throughput Home-Cage-Based Olfactory Discrimination
Source: Front Behav Neurosci. 2021 Jun 9;15:684936. doi: 10.3389/fnbeh.2021.684936 (PMC8219855; doi:10.3389/fnbeh.2021.684936)
Supplement: Supplementary file 1 [file Data_Sheet_1.docx]

# **Supplementary Results**

In addition to p-values, confidence intervals (CI) were calculated to provide a probable range for the effects. 95% confidence intervals were: a) [0.26, 0.50] for the effect of contingency and b) [-0.16, -0.05] for the slope of number of discriminations. The CIs for the slopes correspond to likely ranges for how much the factors (contingency and number of errors) affect number of errors on a logarithmic scale (as data were log-transformed). CIs also indicate statistical significance for the two factors as they do not encompass zero. When confidence intervals were back translated to a linear scale, they correspond to: a) %81 to %218 increase in number of errors for reversal with respect to initial acquisition, b) %10 to %30 reduction in number of errors for each subsequent discrimination.


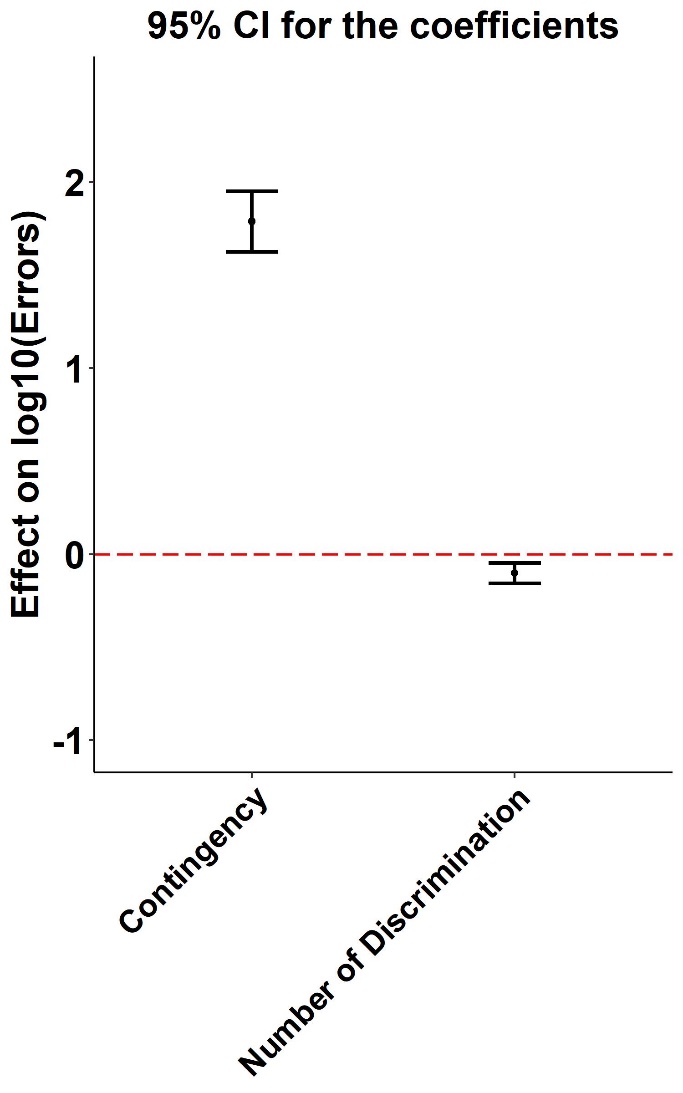


**Fig S1**. Confidence intervals (CIs) for contingency (initial acquisition vs reversal) and number of odor discriminations represented on a logarithmic scale. CIs show the likely magnitude of effects on the number of errors on a logarithmic scale and support statistically significant effects as they do not encompass zero. Backtranslation to the linear scale indicates that those effects correspond to an 81% to 218% increase in number of errors for in the reversal stages compared to the respective discrimination stage as well as a 10% to 30% decrease in number of errors of one discrimination stage compared to the previous discrimination stage.


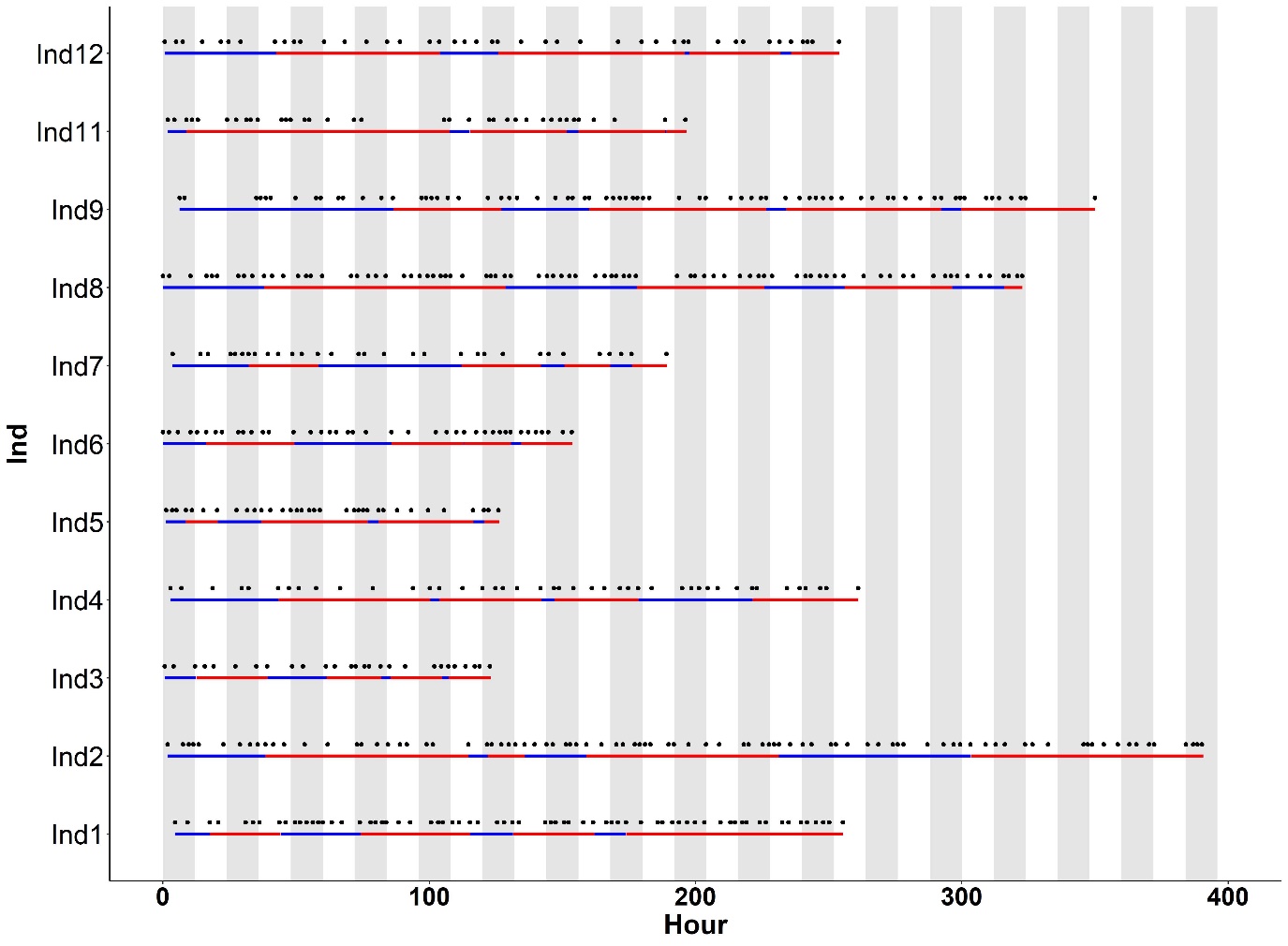


**Fig S2**. Temporal distribution of individual sessions and experimental stages throughout the series of four odor pair discriminations and their reversals. Dark phases indicated by grey bars. Blue shows the initial acquisition stage of an odor pair discrimination and red indicates the subsequent reversal stage for the same odor pair. Data from n=11 mice.


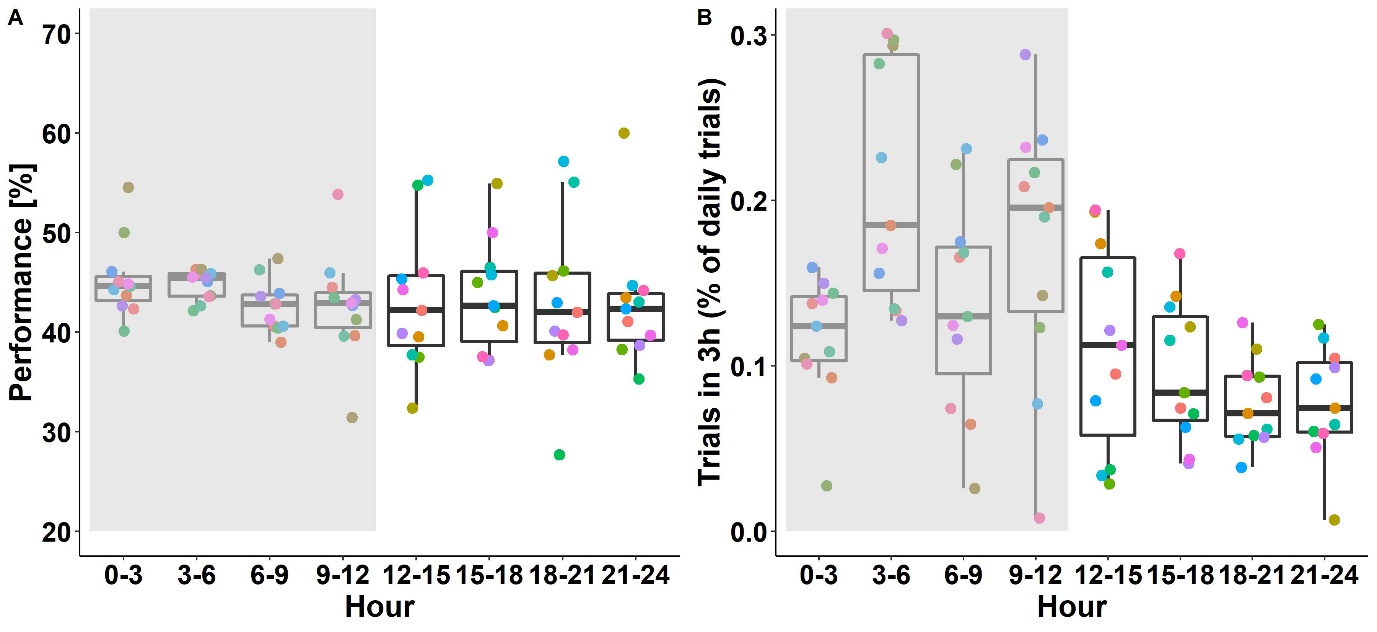


**Fig S3**. Performance and percentage of trials during light and dark phases. Grey rectangles indicate the dark phase. Colored dots represent individual animals. Data combines all trials from odor acquisition and reversals. **A)** Performance of each individual (% correct trials) during the time bins (three hours). As mice advanced immediately after they reached the performance criteria (%85 correct in last 20 trials), mean performance is <50%. **B**) Ratio of trials (with respect to total trials) for each individual mouse during the time bins. Box plots show median, 1^st^ and 3^rd^ quartile, and whiskers the 1.5 interquartile range. Data from n=11 mice

.


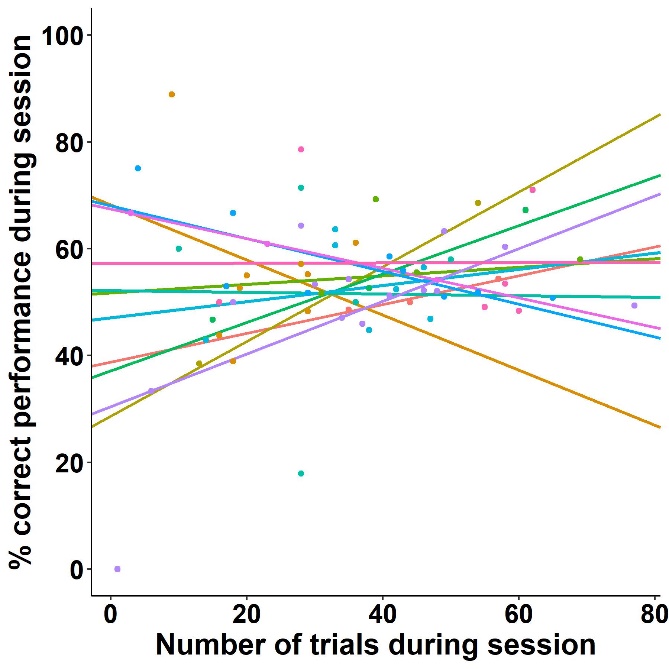


**Fig S4**. The performance level and the number of trials in a session during the first initial odor discrimination acquisition. Dots represent different sessions and color of the dots represent different individuals, while colored lines are linear regression lines fitted for each individual. There is no consistent observable effect of number of trials in a session on performance level. Data from n=11 mice.
